# PathwayBooster: a tool to support the curation of metabolic pathways

**DOI:** 10.1186/s12859-014-0447-2

**Published:** 2015-03-15

**Authors:** Rodrigo Liberal, Beata K Lisowska, David J Leak, John W Pinney

**Affiliations:** Centre for Integrative Systems Biology and Bioinformatics, Department of Life Sciences, Imperial College London, London, SW7 2AZ UK; Department of Biology and Biochemistry, University of Bath, Claverton Down, Bath BA2, 7AY UK

**Keywords:** Metabolic modelling, Model curation, Protein function annotation, Metabolic pathways

## Abstract

**Background:**

Despite several recent advances in the automated generation of draft metabolic reconstructions, the manual curation of these networks to produce high quality genome-scale metabolic models remains a labour-intensive and challenging task.

**Results:**

We present PathwayBooster, an open-source software tool to support the manual comparison and curation of metabolic models. It combines gene annotations from GenBank files and other sources with information retrieved from the metabolic databases BRENDA and KEGG to produce a set of pathway diagrams and reports summarising the evidence for the presence of a reaction in a given organism’s metabolic network. By comparing multiple sources of evidence within a common framework, PathwayBooster assists the curator in the identification of likely false positive (misannotated enzyme) and false negative (pathway hole) reactions. Reaction evidence may be taken from alternative annotations of the same genome and/or a set of closely related organisms.

**Conclusions:**

By integrating and visualising evidence from multiple sources, PathwayBooster reduces the manual effort required in the curation of a metabolic model. The software is available online at http://www.theosysbio.bio.ic.ac.uk/resources/pathwaybooster/.

**Electronic supplementary material:**

The online version of this article (doi:10.1186/s12859-014-0447-2) contains supplementary material, which is available to authorized users.

## Background

The production of a genome-scale metabolic model for any organism is a time-consuming and laborious task [1]. During the various stages of the model curation process there are several bioinformatic resources that can reduce the time required for each stage and have a positive impact on the quality of the resulting model.

The first stage of a genome-scale metabolic reconstruction is the creation of a draft metabolic model. Following the identification and functional annotation of protein-coding genes, comparison of predicted enzymatic functions to a database of known metabolic reactions produces a set of reactions that are presumed to be available to the organism, and hence a network of compounds, reactions and associated enzymes. Resources available for the automated production of a draft genome-scale model include SuBliMinaL Toolbox [2], Model SEED [3] and ERGO [4]. Although automated tools can now produce models that are ready for flux-balance analysis (FBA) [5], these draft metabolic reconstructions are often found to contain numerous inaccuracies [6,7] and require extensive manual curation before they can be considered to be reliable [1].

In the next stages of curation, obvious pathway holes (due to the lack of an assigned enzyme) and false positive reactions (due to enzyme misannotation) need to be found and corrected. To address both of these issues there is a need to collect and analyse evidence for each reaction from the literature and from genomic and metabolic databases, across multiple closely-related species. Without automation this process is tedious and repetitive.

There are already some tools that can tackle this problem allowing comparative analysis of metabolic pathways, such as Comparative Pathway Analyzer [8], FMM [9] and ComPath [10].

Comparative Pathway Analyzer (CPA) [8] is a web implemented tool with the objective of finding the differences in the metabolic networks between two groups of organisms. The maps and reaction annotation data used are taken from the KEGG database. CPA also contains a pathway-reaction display that enables the easy detection of differences between up to six different genome annotations and provides cluster analyses that can include any further annotation uploaded by the user.

FMM [9] is a web server with the prime objective of reconstructing metabolic pathways between two metabolites. It is also mainly based on the KEGG database but integrates other biological databases including UniProtKB/Swiss-Prot [11] and dbPTM [12]. FMM presents the reconstructed pathway by the means of a diagram connecting each of the reactions to information such as metabolites and enzymes involved in the pathway as well as comparative analyses from the species chosen by the user.

ComPath [10] is a complex piece of software that integrates several data sources and tools for pathway analyses and gene annotation in multiple genomes. This information is displayed by means of an interactive spreadsheet, enabling access to several data sources simultaneously. Moreover, it provides tools for structural domain analyses as well as sequence comparison and enzyme prediction.

An ideal piece of software for curating a metabolic model would provide a pathway visualiser together with annotation confidence information and existing literature references. However, none of the packages above contains these features all together.

We have developed PathwayBooster as an open-source software tool to support the comparison and curation of metabolic models. Although other tools exist for the comparative analysis of metabolic pathways, PathwayBooster presents a unique combination of features. Amongst other capabilities, PathwayBooster can be used to compare the functional annotations of genes with ‘bidirectional best BLAST hits’ analyses between the target organism and the relevant related species. It also compiles a list of literature references obtained from BRENDA [13] to support or refute the presence of each enzyme within the selected species. An interactive graphical summary of the evidence found in each organism is produced in the form of a clickable KEGG pathway diagram.

## Implementation

PathwayBooster is implemented in Python and can either be used as an command-line tool or through a graphical interface. The user supplies input in the form of GenBank, EMBL or FASTA files for all the organisms that are to be compared. Output is presented as a browsable set of HTML files, with sections that are described in more detail below. Instructions on how to run PathwayBooster can be found in the user manual (see Additional file 1).

One of the key advantages of PathwayBooster is in the use of KEGG API. This is a web service allowing access to the KEGG database in an automated way using a REST interface. In this way, PathwayBooster always provides up-to-date KEGG data.

**Figure 1:**
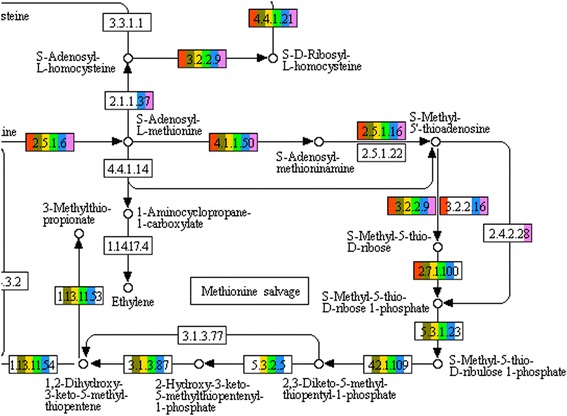
**Pathway diagram example.** Detail from an example pathway diagram produced by PathwayBooster, showing methionine salvage (a part of cysteine and methionine metabolism). The coloured blocks show an automated model produced by ERGO^™^ for the thermophilic bacterium *G. thermoglucosidasius* NCIMB 11955 (red) in comparison to selected reference organisms: *G. thermoglucosidasius* C56-YS93 (brown), *G. kaustophilus* (yellow), *G.thermodenitrificans* (green), *B. subtilis* (blue) and *E. coli* (purple).

Using REST to access KEGG’s pathway templates, PathwayBooster returns an interactive image where all reactions are colour coded according to the presence or absence of a given reaction in each chosen species (Figure 1). In the KEGG pathway display, information about each reaction can be accessed via a popup menu showing the available options for a given enzyme. Information is divided into three groups: annotations, BLAST results and literature. Each choice can be accessed by its own hyperlink, redirecting the user into a new window where the corresponding data can be viewed. All functions can also be accessed through the tabs in the top of the pathway image. However, the use of the popup menu will restrict the report data in each different group to the enzymatic function specified.

### Annotations

The annotation table is divided according to the Enzyme Commission (EC) numbers present in a pathway of interest. Annotated genes are presented by EC number for all specified organisms. Each gene is hyperlinked to the KEGG database, where associated information can be viewed. It also indicates the origin of each annotation. This is relevant when more than one genome annotation source is under consideration. With the exception of KEGG, all annotation sources must be supplied by the user. In the case of KEGG annotations the data is retrieved using the REST web service as before.

### BLAST results

Two proteins from two different organisms are called ‘best reciprocal hits’ when each is the best BLAST hit of the other. This is a simple method commonly used to find putative orthologous proteins, i.e. proteins descending from a common ancestor that have diverged following a speciation event [14]. These proteins tend to have similar sequences and are likely to have similar functions. Evidence from best reciprocal hits can be very helpful in the curation of a metabolic model with respect to a related, well-annotated reference genome. It can be used either to support a given functional annotation or to find a candidate protein for a missing function. Based on the genome information provided by the user, BLAST [15] best reciprocal hits are made available in PathwayBooster for a selected ‘query’ organism compared against the other species supplied by the user. Each protein hit is followed by its annotated function, the corresponding EC number and the sequence similarity, E-value and Z-score for the alignment between the two proteins.

To find possible candidate proteins for a particular function, the first three BLAST hits from the ‘query’ genome can also be viewed for every enzyme annotated in the reference species. This report also provides the functional annotation and EC number for each candidate, as well as the sequence similarity, E-value and Z-score as before.

### Literature

PathwayBooster makes use of the BRENDA database to provide information about publications connecting a given organism with a particular enzymatic function. For each pathway selected, publications from BRENDA that assert the presence of each EC number in each specified organism are listed. Publications indicating that a given EC number might be absent in an organism are also available. Each publication has a hyperlink to the PubMed website, where its abstract can be viewed. The number of manually annotated references available in BRENDA is currently over 100,000 [13].

### Heat map

For a given KEGG pathway, we can define a Hamming distance between two organisms as the number of enzymatic functions present in one but not both of those organisms. In the PathwayBooster report a heat map is provided to show the Hamming distance between the organisms selected, according to the presence or absence of each enzyme in the pathway. This simple visualisation of the similarity between pathway structures can be used to support comparative analysis or to summarise the relative consistency of different annotation sources.

## Results and discussion

This section presents examples from the curation of a genome-scale metabolic model where the advantages of using PathwayBooster are clearly seen.

*Geobacillus thermoglucosidasius* NCIMB 11955 is a thermophilic bacterium with the potential to convert lignocellulose to ethanol in a highly productive manner. Thermophilic bacteria are especially useful in biofuel production since they can withstand the high temperatures that are unavoidable at certain stages of fermentation. Given these interesting properties, we would like to understand the metabolism of this organism in more detail.

As an example, PathwayBooster results for cysteine and methionine metabolism (KEGG pathway 00270) are presented. The initial draft metabolic network was built using ERGO [4]. Reference organisms for comparison in PathwayBooster were selected to include well-studied bacterial genomes (*Escherichia coli*, *Bacillus subtilis*), other species within the same genus as the target organism (*Geobacillus thermodenitricans*, *Geobacillus kaustophilus*) and a different strain of the same species (*Geobacillus thermoglucosidasius* C56-YS9). Evidence for the presence of enzymes in these comparison genomes was retrieved from KEGG. In addition, BLAST analysis of the query organism was carried out against the *E. coli* and *B. subtilis* annotated proteomes.

### Filling pathway holes

**Figure 2:**
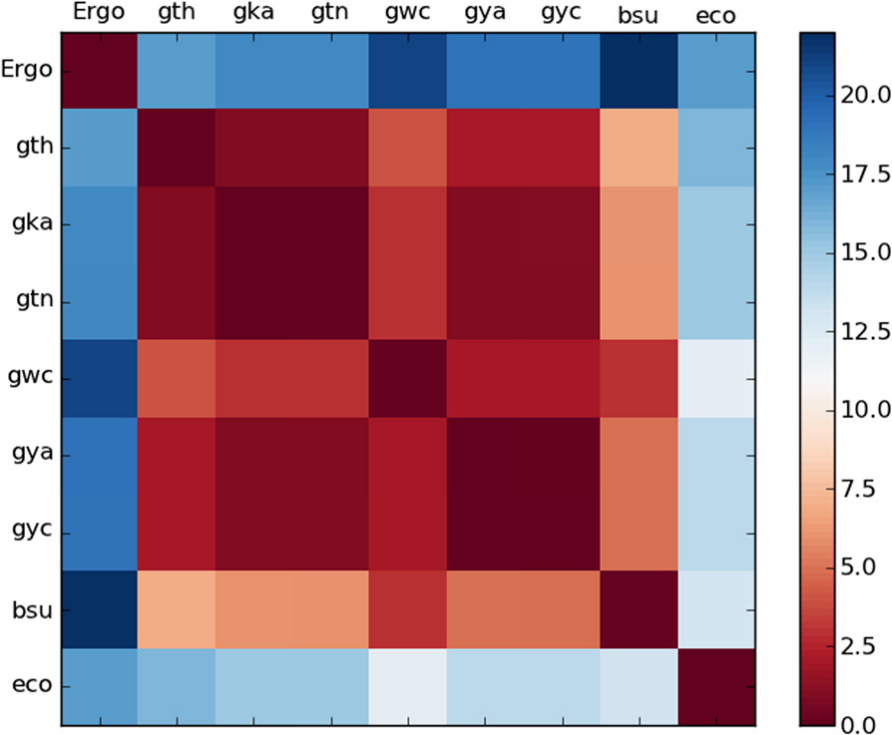
**Hamming distance heatmap for cysteine and methionine metabolism.** Hamming distance heatmap for cysteine and methionine metabolism, showing the similarity between the query species (marked ‘Ergo’) and reference organisms.

The Hamming distance heatmap (Figure 2) gives us the first evidence of an unexpected difference between the *Geobacillus thermoglucosidasius* draft metabolic network and the the other organisms. Examining the pathway diagram (Figure 1), it can easily be seen that the reactions tagged with the EC numbers 4.2.1.109, 3.1.3.77, 1.13.11.53 and 5.3.1.23 are not annotated for the query organism, in contrast to most of the reference organisms. A possible explanation is that the enzymes with these functions were not identified by the ERGO annotation servers.

**Figure 3:**
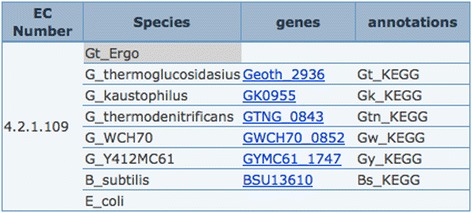
**General information.** General information for EC 4.2.1.109 (5-methylthioribulose-1-phosphate dehydratase).

Making use of the PathwayBooster publication tables for each function present in the pathway, an article can be found relating to the enzyme 4.2.1.109 (5-methylthioribulose-1-phosphate dehydratase) in *Bacillus subtilis* [16]. The article referenced is easily accessed by clicking in the hyperlink provided in the table. For each genome considered, proteins annotated for each function can be found in the ‘Annotations’ report. This table provides easy access to further information for each gene via the KEGG database (Figure 3).

**Figure 4:**
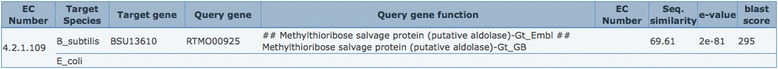
**BLAST bidirectional best hit.** BLAST bidirectional best hit for EC 4.2.1.109 (5-methylthioribulose-1-phosphate dehydratase).

**Figure 5:**
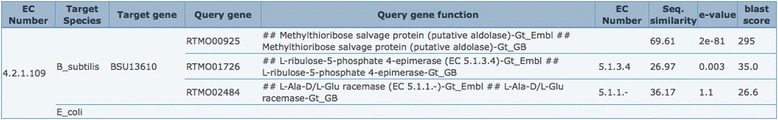
**Three best BLAST hits.** Three best BLAST hits for EC 4.2.1.109 (5-methylthioribulose-1-phosphate dehydratase).

To find candidates for filling the enzymatic function 4.2.1.109, PathwayBooster’s ‘BLAST bidirectional hits’ report was used to retrieve a promising candidate gene within the *G. thermoglucosidasius* NCIMB 11955 genome (Figure 4) with significant similarity to the *B. subtilis* enzyme confirmed in [16]. For a less stringent search, PathwayBooster’s ‘Three BLAST hits’ report retrieves the three best BLAST hits for each gene against the query genome. Each hit also reports the sequence similarity information, E-value and Z-score (Figure 5).

The procedure described was also successfully applied to the remaining missed annotations, finding candidate genes for each of them.

### Identifying misannotated enzymes

In contrast to the example shown above, the enzyme function 5’-methylthioadenosine nucleosidase (EC 3.2.2.16) was found in the annotation of the query strain and not found in the closely related reference organisms. The most probable explanations are that either the gene annotated with this enzymatic function has been wrongly assigned, or that *G. thermoglucosidasius* has acquired a new function that is not present in its close relatives.

By examining the ‘Publications’ reports, this function is not found in any of the relevant literature. Taking a closer look at the assigned gene, RTMO02286, in the ‘Annotations’ section, we see that the gene has been assigned with two potential functions: 5-methylthioadenosine nucleosidase (EC 3.2.2.16) and S-adenosylhomocysteine nucleosidase (EC 3.2.2.9). All of the reference organisms have an annotation for EC 3.2.2.9 and this function is also supported by the ‘BLAST hits’ report. Therefore, it was concluded that EC 3.2.2.16 is most likely to be a misannotation and that the most probable function annotation for RTMO02286 is EC 3.2.2.9.

## Conclusions

Resources such as Model SEED [3] can be used to produce draft metabolic models, but are not designed to support further model curation. PathwayBooster provides a single integrated interface to literature references, BLAST evidence and annotations from alternative sources or related organisms. Most importantly, PathwayBooster provides a logical visual representation of its results, significantly reducing the effort needed to identify enzyme misannotations and pathway holes. The information provided by PathwayBooster can be particularly useful when working with a platform for genome-scale model curation such as MEMOSys [17] or GEMSiRV [18]. Although several other tools exist to support comparative pathway analysis, PathwayBooster provides a unique combination of features that make it particularly suitable for use in model curation.

## Availability and requirements

- **Project name**: PathwayBooster
- **Project homepage**: http://www.theosysbio.bio.ic.ac.uk/resources/pathwaybooster/
- **Operating systems**: Linux, Mac OSX, Windows.
- **Other requirements**: BRENDA flatfile database (available from http://www.brenda-enzymes.org/, free for academic use)
- **Programming language**: Python
- **License**: GPLv3

## Additional file

Additional file 1
**PathwayBooster manual.**
