# Supplementary material for: PathwayBooster: a tool to support the curation of metabolic pathways
Source: BMC Bioinformatics. 2015 Mar 15;16(1):86. doi: 10.1186/s12859-014-0447-2 (PMC4367891; doi:10.1186/s12859-014-0447-2)
Supplement: Additional file 1 — PathwayBooster manual. [file 12859_2014_447_MOESM1_ESM.pdf]

PathwayBooster

Rodrigo Liberal, Beata K. Lisowska, David J. Leak and John W. Pinney

June 2014

1 Introduction

PathwayBooster is an open-source software tool to support the comparison and curation of metabolic models. It combines gene annotations from GenBank files and other sources with information retrieved from the metabolic databases BRENDA and KEGG to produce a set of pathway diagrams and reports summarising the evidence for the presence of a reaction in a given organism's metabolic network. By comparing multiple sources of evidence within a common framework, PathwayBooster assists the curator in the identification of likely false positive (misannotated enzyme) and false negative (pathway hole) reactions. Reaction evidence may be taken from alternative annotations of the same genome and/or a set of closely related organisms.

This document provides information on how to install and run PathwayBooster. The software has been built and tested with Python 2.6 and newer, on Windows, Mac OS X, and Linux platforms. It is also available a PathwayBooster version with a graphical interface which is only available for windows.

PathwayBooster may be downloaded from
`http://www.theosysbio.bio.ic.ac.uk/resources/pathwaybooster/`.

For support and other queries, send e-mail to j.pinney@imperial.ac.uk.

2 Setup instructions

2.1 Prerequisites

- Python[1]. Tested with versions 2.6+.
- BLAST[2]. Tested with version 2.2.24.
- PIL[3] python modules. If you encounter problems installing PIL or when running PathwayBooster, with an error like '`ImportError: The _imagingft C module is not installed`', you should try installing a PIL version with precompiled libraries[4]. Using Enthought python, PIL should already be installed.
- BRENDA flatfile[5]. Once extracted from the zipfile, move the file `brenda_download.txt` into the `PathwayBooster/files` directory.

2.2 Installation terminal version

This section describes how to install PathwayBooster and get it working.

Download `PathwayBooster-<version>`.zip from `http://www.theosysbio.bio.ic.ac.uk/resources/pathwaybooster/` and unzip.

Download the BRENDA flatfile (freely obtainable from `http://www.brenda-enzymes.info/`) and unpack. Save this in the `PathwayBooster/files` directory as `brenda_download.txt`

`NOTE:` you will have to register to download the Brenda file

Make a setup file as explained bellow in the `Setup File` section and save it in your working directory

- `Running PathwayBooster`

  To run PathwayBooster type:

  `python PathwayBooster.py [setupFilename.xml]`

  Since BLAST runs may take a while, there is the option of pre-compiling the BLAST files without running the other analyses. To do so, type:

  `python PathwayBooster.py -blast [setupFilename.xml]`

[1]e.g. `http://enthought.com/repo/free/`
[2]`ftp://ftp.ncbi.nlm.nih.gov/blast/executables/blast+/`
[3]`http://www.pythonware.com/products/pil/`
[4]available from `http://www.lfd.uci.edu/~gohlke/pythonlibs/`
[5]`http://www.brenda-enzymes.org/brenda_download/`

By default, PathwayBooster saves the results in a directory named `PathwayBoosterReports`. To change the output directory, type:

`python PathwayBooster.py [setupFilename.xml] -outDir [newOutPutDirectory]`

PathwayBooster does not need to run from the PathwayBooster directory. To run from a different directory, just type:

`python path/to/PathwayBooster.py [setupFilename.xml]`

and the results will be saved in the current working directory.

2.3 Installation GUI version

This section describes how to install PathwayBoosterGUI and get it working. The GUI version is only available for Windows.

Download `PathwayBoosterGUI-<version>.zip` from `http://www.theosysbio.bio.ic.ac.uk/resources/pathwaybooster/` and unzip.

Download the BRENDA flatfile (freely obtainable from `http://www.brenda-enzymes.info/`) and unpack. Save this in the `PathwayBooster/files` directory as `brenda_download.txt`

To start PathwayBoosterGUI double click on PathwayBoosterGUI.exe file.

Instructions on how to use PathwayBoosterGUI can be found bellow and in the help support given in PathwayBoosterGUI.

2.4 Setup File

The setup file is constructed in XML format and is divided into three parts that reflect groups of information to be provided by the user: `<pathwayList>`, `<genomeList>` and, optionally, `<blockList>`. An example setup file is shown in Fig. 2.1.

<pathwayList>

In this section, the user specifies the set of KEGG pathways to be processed as a series of `<pathway>` elements. There are two ways to specify pathways:

- using KEGG metabolic function groups[6], e.g. carbohydrate metabolism has id 1.1. The declaration `<pathway id=1.1>` means that all pathways in this group are processed.
- using the global KEGG id of an individual pathway, e.g. `<pathway id = 00010>` corresponds to Glycolysis/Gluconeogenesis.

<genomeList>

This section requires the user to specify genome information for the species of interest and other reference organisms. For each organism to be included, the user must define a `<genome>` element. The attribute `name` refers to a species identifier, which will be used by the software for display. For each `<genome>`, the user may provide multiple `<annotation>`

[6] `http://www.kegg.jp/kegg/pathway.html`

sources. These can be of three different kinds, defined by the attribute `type`: `kegg`, `genbank` or `embl`. For `genbank` and `embl`, the user must provide a `filename` for a genome annotation in the respective file format. For the `kegg` annotations, the user provides the `keggId` for the given genome. For example, in the case of *Bacillus subtilis*, set `keggId=bsu`. The user can provide more than one annotation of each type, however all the annotations must have a unique `id` attribute.

For each `<genome>` there are multiple options available, specified by the following attributes:

- `filename`
  The user may supply a FASTA-format file containing amino acid sequences for the predicted proteome.
- `query`
  When set to `true`, this signifies that this genome is the one of main interest. If none of the genomes is set with `query=true`, the first genome with a genome annotation sequence file provided will be considered as the query genome.
- `brenda`
  The full taxonomic name of the organism, which will be used by PathwayBooster to search the BRENDA database in order to retrieve publication information.
- `color`
  The color that should be used to identify the genome in the PathwayBooster display. The accepted format is the RGB color model. This format is constituted by 3 numbers between 0 and 255 separated by commas. An example would be `color="30,40,200"`. If the user does not specify a color, PathwayBooster will attribute one automatically.
- `pathway`
  This controls whether the genome is included in the pathway visualisation. (Default is `true`). There can be a maximum of 7 genomes displayed. A reaction is considered as present if it has either an annotated gene (from any of the annotations provided) or literature evidence (if `brenda` is provided).
- `hamming`
  This controls whether the genome is included in the Hamming distance matrix. (Default is `true`).

`<blockList>`

This optional section can be used to specify more complex display preferences, for example if the user wants to compare the annotations that were obtained from two different sources for the same organism, these can be separated into different `<block>` elements. All options available for a `<genome>` are also available for a `<block>`, with the exception of `filename`. When the `<blockList>` section is present, the `<genome>` attributes will be overridden for the pathway map, Hamming distance and literature evidence displays.

Within each `<block>` element, the user specifies one or more `<annotationReference>` elements, with an `id` matching that of an `<annotation>` specified previously. By choosing annotations from multiple organisms, it is possible to compare groups of genomes against the query organism.

```
<xml>

<pathwayList>
       <pathway id="00270"/>
</pathwayList>

<genomeList>
<genome name="Gt_Ergo" filename="ERGO/TMO_protein.txt">
       <annotation type="embl" id="Gt_Embl" filename="ERGO/TMO_embl.txt"/>
       <annotation type="genbank" id="Gt_GB" filename="ERGO/TMO_genbank.txt"/>
</genome>
<genome name="G_thermoglucosidasius" brenda="Geobacillus thermoglucosidasius">
       <annotation type="kegg" id="Gt_KEGG" keggId="gth"/>
</genome>
<genome name="G_kaustophilus" brenda="Geobacillus kaustophilus">
   <annotation type="kegg" id="Gk_KEGG" keggId="gka"/>
</genome>
<genome name="G_thermodenitrificans" brenda="Geobacillus thermodenitrificans">
       <annotation type="kegg" id="Gtn_KEGG" keggId="gtn"/>
</genome>
<genome name="G_WCH70" brenda="Geobacillus sp. WCH70" pathway="false">
       <annotation type="kegg" id="Gw_KEGG" keggId="gwc"/>
</genome>
<genome name="G_Y412MC61" brenda="Geobacillus sp. Y412MC61" pathway="false">
       <annotation type="kegg" id="Gy_KEGG" keggId="gyc"/>
</genome>
<genome name="B_subtilis" brenda="Bacillus subtilis" filename="PepSeq/b.subtilis.pep">
       <annotation type="kegg" id="Bs_KEGG" keggId="bsu"/>
</genome>
<genome name="E_coli" brenda="Escherichia coli" filename="PepSeq/e.coli.pep">
       <annotation type="kegg" id="Ec_KEGG" keggId="eco"/>
</genome>
</genomeList>


<blocklist>
<block name="Gt_Ergo_Embl" query="true" color="255,0,0" pathway="true" filename="ERGO/TMO_protein.txt">
       <annotationReference id="Gt_Embl"/>
</block>
<block name="Gt_Ergo_GB" color="0,255,0" pathway="true">
   <annotationReference id="Gt_GB"/>
</block>
<block name="Gt_Kegg" color="0,0,255" pathway="true">
       <annotationReference id="Gt_KEGG"/>
</block>
<block name="Gt_Kegg_pub" color="100,0,100" pathway="true" brenda="Geobacillus thermoglucosidasius">
</block>
<block name="G_WCH70_Y412MC61" pathway="false">
       <annotationReference id="Gk_KEGG"/>
       <annotationReference id="Gtn_KEGG"/>
</block>
<block name="G_kaust" pathway="true" brenda="Geobacillus kaustophilus">
       <annotationReference id="Gk_KEGG"/>
</block>
<block name="G_thermo" pathway="true" brenda="Geobacillus thermodenitrificans">
       <annotationReference id="Gtn_KEGG"/>
</block>
<block name="Bs_Ec" pathway="true">
       <annotationReference id="Bs_KEGG"/>
       <annotationReference id="Ec_KEGG"/>
</block>
<block name="B_sub" pathway="false" hamming="false" brenda="Bacillus subtilis" filename="PepSeq/b.subtilis.pep">
              <annotationReference id="Bs_KEGG"/>
</block>
<block name="E_co" pathway="false" hamming="false" brenda="Escherichia coli" filename="PepSeq/e.coli.pep">
              <annotationReference id="Ec_KEGG"/>
</block>
</blocklist>

</xml>
}
```

Figure 2.1: Setup file example for PathwayBooster.

2.5 Graphical interface manual

This section provides a detailed description of PatwayBooster GUI. PathwayBooster GUI is formed by 4 main sections organised in tabs.

- `Preferences`
- `Organisms`
- `Pathways`
- `Run`

The objective of PatwayBooster GUI is to help the user to build the xml setup file (for more details, see section 2.3) and define some preferences.

- **Preferences**

  In this tab the user can define some preferences such as where the report will be saved and its name.

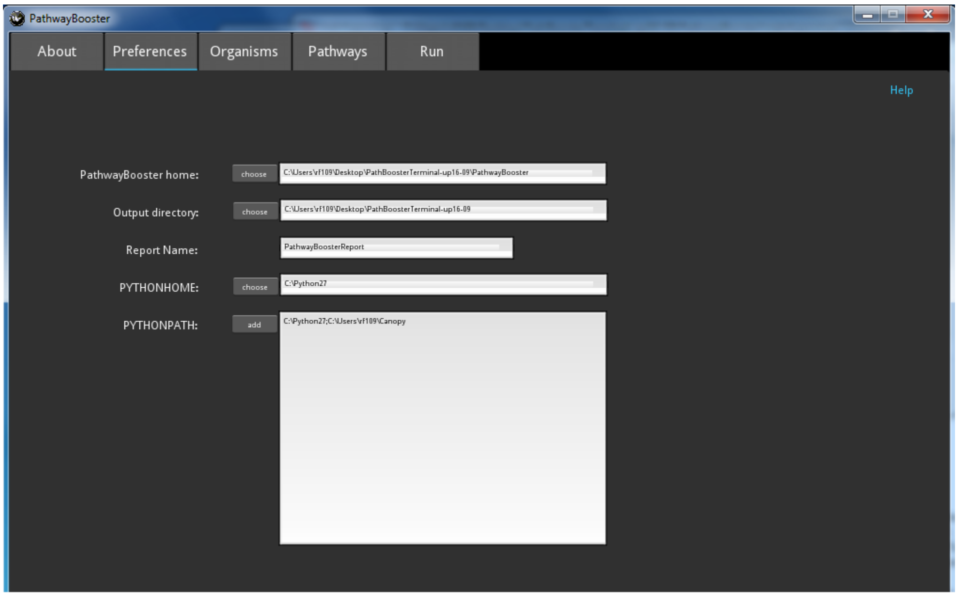

Figure 2.2: Preferences Tab. `PathwayBooster home:` Specify where the annotation files are. This will make the task of finding the annotation files easier and faster; `Output directory:` Specify the directory where you want to save the PathwayBooster Report. By default the report will be saved in the same directory as PathwayBooster; `Report Name:` Indicate the name of the PathwayBooster Report. By default the name is PathwayBoosterReport; `PYTHONHOME:` Indicate the directory that contains the python "Lib" folder.; `PYTHONPATH:` Indicate the directories that contain the python modules needed to run PathwayBooster.

- **Organisms**

  This tab relates to the `genomeList` section of the xml setup file. Here, the user will have to specify genome information for the species of interest and other reference organisms.

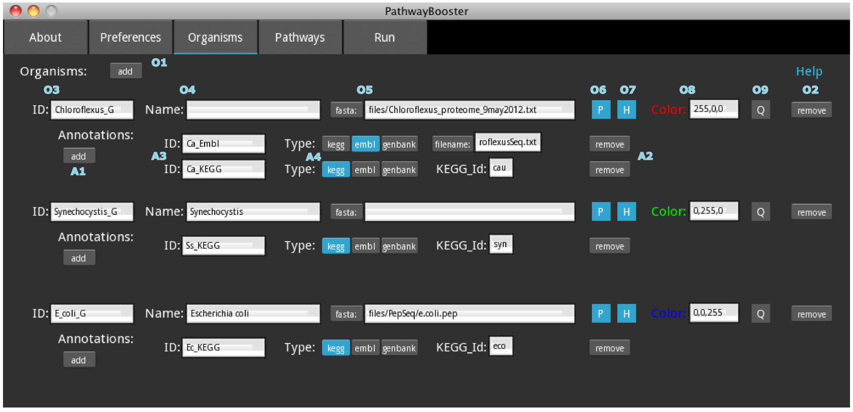

Figure 2.3: Organims Tab. `01 - add:` Adds an organims object to the display; `02 - remove:` Removes that specific organims; `03 - ID:` Refers to the organism identifier, which will be used by the software for display. Each ID must be unique; `04 - Name:` The full taxonomic name of the organism, which will be used by PathwayBooster to search the BRENDA database in order to retrieve publication information; `05 - fasta:` The user may supply a FASTA-format file containing amino acid sequences for the predicted proteome. By clicking in the fasta button a popup window with a file-handling menu will appear; `06 - P:` This controls whether the genome is included in the pathway visualisation. (Default is true - blue background). There can be a maximum of 7 genomes displayed. A reaction is considered as present if it has either an annotated gene (from any of the annotations provided) or literature evidence (if brenda is provided); `07 - H:` This controls whether the genome is included in the Hamming distance matrix. (Default is true - blue background); `08 - Color:` The color that should be used to identify the genome in the PathwayBooster display. It accepts 3 different formats:RGB color model - 3 numbers between 0 and 255 separated by commas (Ex:255,102,0); RGB color model - 3 numbers between 0 and 1 separated by commas (Ex:1,0.4,0); hexadecimal color model - starts with a # foloowed by 3 hexadecimal number between 00 and FF (Ex:#FF6700). If the user does not specify a color, PathwayBooster will attribute one automatically; `09 - Q:` When set to true (blue background), this signifies that this genome is the one of main interest. If none of the genomes is set with query=true, the first genome with a genome annotation sequence file provided will be considered as the query genome; `A1 - add:` Adds an annotation object to the organism; `A2 - remove:` Removes that specific annotation; `A3 - ID:` Refers to the organism identifier. Each ID must be unique; `A4 - Type:` These can be of three different kinds: kegg, genbank or embl. For the kegg annotations, the user provides the kegg_Id for the given genome. For genbank and embl, the user must provide a filename for a genome annotation in the respective file format. Avoid typing the pathway, use the file handler provided when pressing the filename button.

- **Pathways**

  In this section, the user specifies the set of KEGG pathways to be processed as a series of `<pathway>` elements in the xml setup file (see manual for more details). There are two ways to specify pathways:

  – using KEGG metabolic function groups, e.g. carbohydrate metabolism has id 1.1. This means that all pathways in this group are processed.

  – using the global KEGG id of an individual pathway, e.g. 00010 corresponds to Glycolysis/Gluconeogenesis.

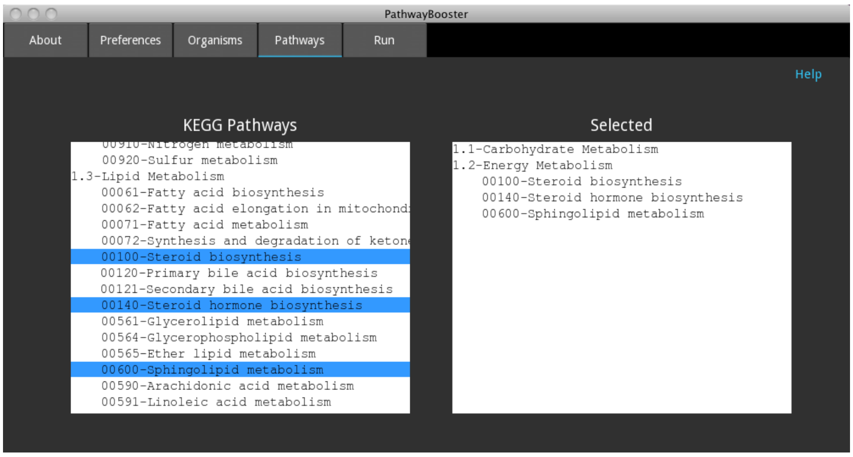

Figure 2.4: Pathway Tab. `KEGG Pathways:` In this sliding window are present all the KEGG metabolic pathways. To select a pathway the just needs to click on it. All the selected pathways appear in the Selected sliding window and have a blue background; `Selected:` All the selected Pathways will appear here. To remove any of the pathways selected, the user just needs to click on it.

- **Run**

  In this section the user can run the PathwayBooster tool with the settings showing in the Pathways and Organisms tabs. In this section the user also has the opportunity to import any previously built setup file or save into an xml file the settings showing in the Pathway and Organisms Tabs.

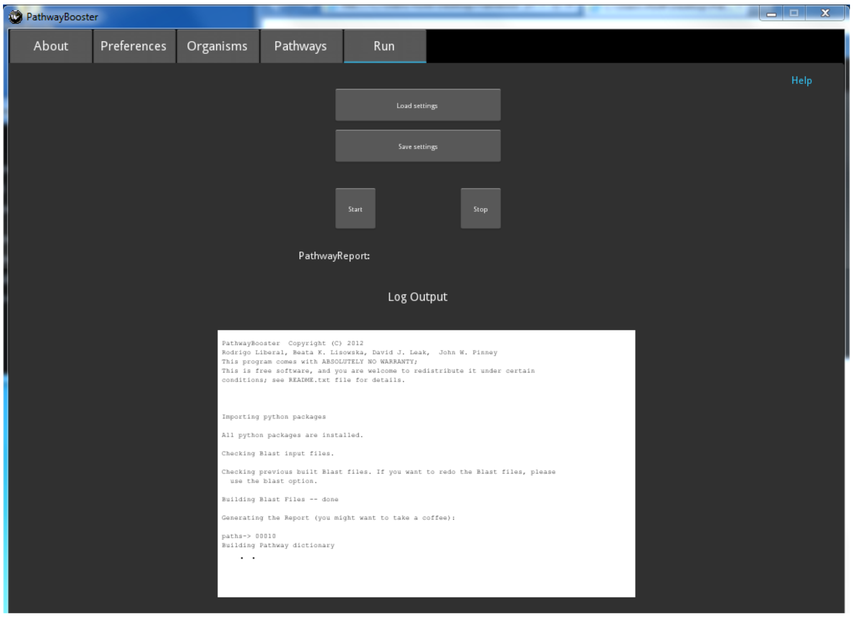

Figure 2.5: Pathway Tab. `Load settings:` Load an xml setup file with PathwayBooster settings. After import the file the settings can be seen in the Organisms and Pathways tabs; `Save settings:` Save to a file the settings present in the Organisms and Pathways sections; `Start:` Starts PathwayBooster using the settings present in the Organisms and Pathways sections; `Stop:` Stop the PathwayBooster process. This button is only present while PathwaysBooster is running; `Output Messages:` Save to a file the settings present in the Organisms and Pathways sections; `PathwayReport:` After a successful run the link present here will open the PathwayBooster report.

2.6 PathwayBooster Report Index

After running PathwayBooster, a report will be produced. The index page will contain links to every pathway requested. Figure 2.6 shows an example of a possible index page. In this case, the user requested all the *Energy metabolism* pathways and two other pathways grouped in the *Other Pathways* section. The links provided for each pathway will contain the information described in the paper and in the *Example Application* bellow.

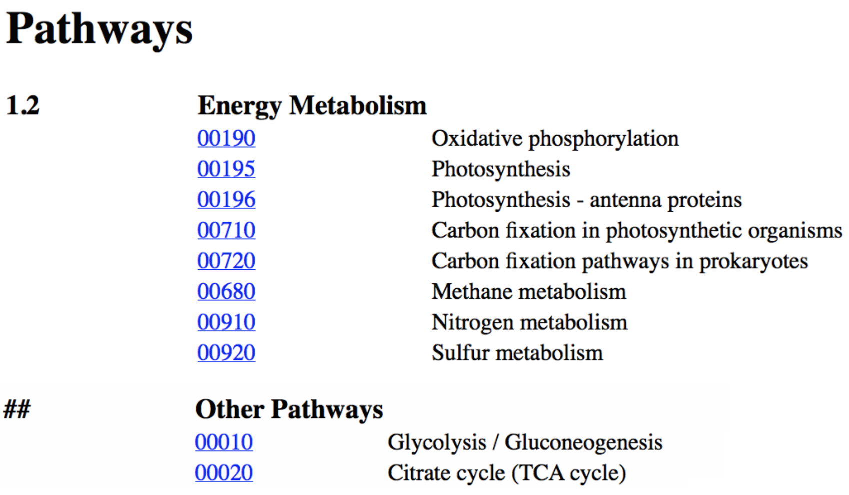

Pathways

1.2	**Energy Metabolism**	
	00190	Oxidative phosphorylation
	00195	Photosynthesis
	00196	Photosynthesis - antenna proteins
	00710	Carbon fixation in photosynthetic organisms
	00720	Carbon fixation pathways in prokaryotes
	00680	Methane metabolism
	00910	Nitrogen metabolism
	00920	Sulfur metabolism
##	**Other Pathways**	
	00010	Glycolysis / Gluconeogenesis
	00020	Citrate cycle (TCA cycle)

Figure 2.6: PathwayBooster Report index example.

3 Example Application

Geobacillus thermoglucosidasius NCIMB 11955 is a thermophilic bacterium with the potential to convert lignocellulose to ethanol in a highly productive manner. Thermophilic bacteria are especially useful in biofuel production since they can withstand the high temperatures that are unavoidable at certain stages of fermentation. Given these interesting properties, we would like to understand the metabolism of this organism in more detail.

As an example of the use of PathwayBooster, we present results for cysteine and methionine metabolism (KEGG id = 00270). Initial genome annotations were generated by ERGO™ integrated genomics (Overbeek *et al.* (2003)) and the RAST annotation server (Aziz *et al.* (2008)). The agreements and differences between these annotations were used in the first stages of metabolic model curation for this organism. At a later stage, reference organisms were selected, including *Escherichia coli*, *Bacillus subtilis*, *Geobacillus thermoglucosidasius* C56-YS93, *Geobacillus thermodenitrificans* and *Geobacillus kaustophilus.*

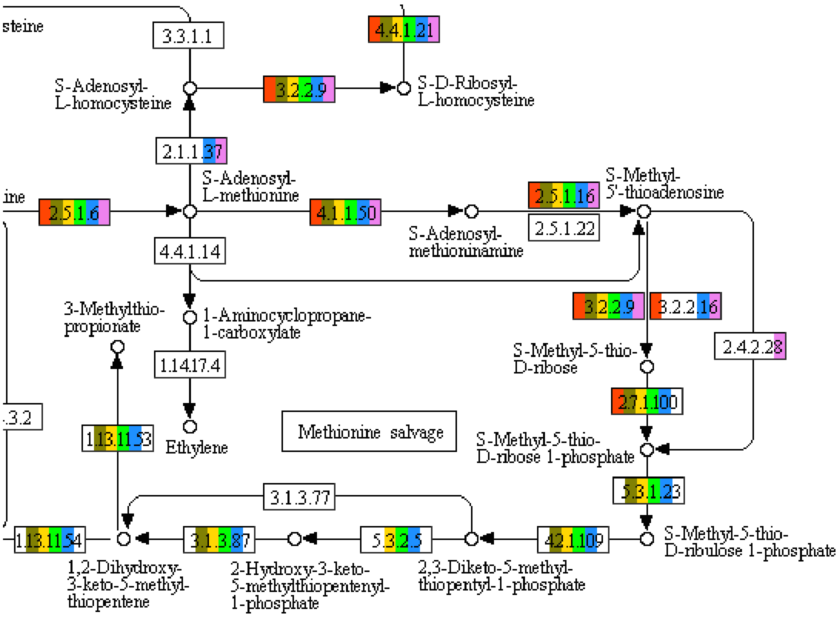

Figure 3.1: Visual representation of the methionine salvage pathway, where *G. thermoglucosidasius* NCIMB 11955 (red) is compared to selected reference organisms: *G. thermoglucosidasius* C56-YS93 (brown), *G. kaustophilus* (yellow), *G.thermodenitrificans* (green), *B. subtilis* (blue) and *E. coli* (purple).

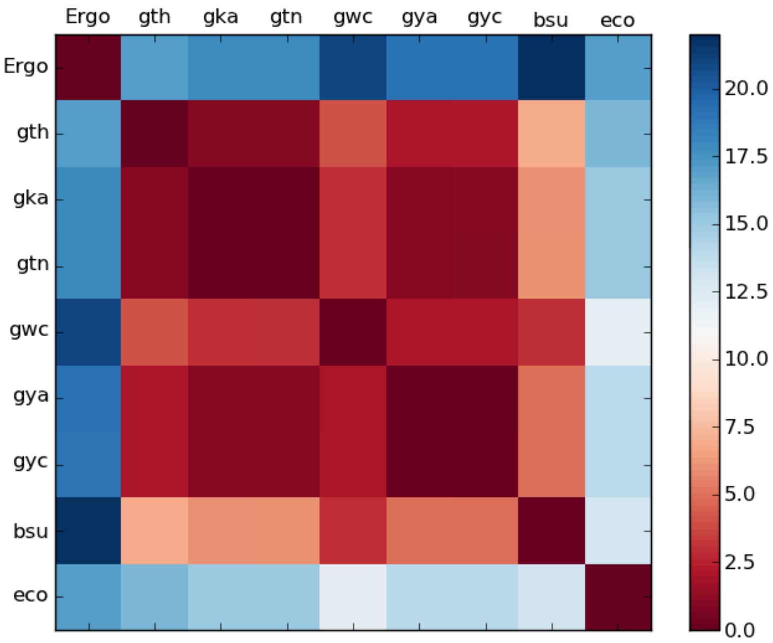

Figure 3.2: Hamming distance heatmap for cysteine and methionine metabolism, showing the similarity between the query species (marked 'Ergo') and reference organisms.

3.1 Filling pathway holes

Looking at the visual representation of this pathway (Fig 3.1) and the Hamming distance heatmap (Figure 3.2) generated by PathwayBooster, it can be observed that enzymes with the EC numbers 4.2.1.109, 3.1.3.77, 1.13.11.53 and 5.3.1.23 are not annotated for the query strain (red), but are present in most of the reference organisms. It is possible that these enzymes were missed by the ERGO/RAST annotation servers.

Using PathwayBooster we looked at the publications for the omitted enzymes, retrieving a relevant article with information on enzyme 4.2.1.109 (5-methylthioribulose-1-phosphate dehydratase) in *Bacillus subtilis* (Ashida *et al.* (2008)).

The article was easily accessible via its Pubmed accession number. Information on enzyme mass and activity will be used in the design of laboratory experiments, but the retrieved gene sequence was immediately useful for finding similar genes within the genome of *G. thermoglucosidasius* NCIMB 11955.

EC Number	Species	papers	pubmed number
4.2.1.109	B_subtilis	Ashida, H.; Saito, Y.; Kojima, C.; Yokota, A.: Enzymaticcharacterization of 5-methylthioribulose-1-phosphate dehydratase of themethionine salvage pathway in Bacillus subtilis. Biosci. Biotechnol.Biochem. (2008) 72, 959-967.	Pubmed:18391471

Sequences for all relevant genes from related organisms can be easily accessed from the 'Annotations' report in PathwayBooster. Here, hyperlinks redirect the user to the KEGG website (Kanehisa *et al.* (2012, 2000)) where information on a gene, including its nucleotide and amino acid sequence can be accessed:

EC Number	Species	genes	annotations
4.2.1.109	Gt_Ergo		
	G_thermoglucosidasius	Geoth_2936	Gt_KEGG
	G_kaustophilus	GK0955	Gk_KEGG
	G_thermodenitrificans	GTNG_0843	Gtn_KEGG
	G_WCH70	GWCH70_0852	Gw_KEGG
	G_Y412MC61	GYMC61_1747	Gy_KEGG
	B_subtilis	BSU13610	Bs_KEGG
	E_coli		

Using the 'BLAST bidirectional hits' report, a candidate gene within *G. thermoglucosidasius* NCIMB 11955 genome was identified:

EC Number	Target Species	Target gene	Query gene	Query gene function	EC Number	Seq. similarity	e-value	blast score
4.2.1.109	B_subtilis	BSU13610	RTMO00925	## Methylthioribose salvage protein (putative aldolase)-Gt_Embl ## Methylthioribose salvage protein (putative aldolase)-Gt_GB		69.61	2e-81	295
	E_coli							

It is also possible for the user to view the top three BLAST hits for the model organism, accompanied by the sequence similarity information, e-value and overall BLAST score:

EC Number	Target Species	Target gene	Query gene	Query gene function	EC Number	Seq. similarity	e-value	blast score
4.2.1.109	B_subtilis	BSU13610	RTMO00925	## Methylthioribose salvage protein (putative aldolase)-Gt_Embl ## Methylthioribose salvage protein (putative aldolase)-Gt_GB		69.61	2e-81	295
			RTMO01726	## L-ribulose-5-phosphate 4-epimerase (EC 5.1.3.4)-Gt_Embl ## L-ribulose-5-phosphate 4-epimerase-Gt_GB	5.1.3.4	26.97	0.003	35.0
			RTMO02484	## L-Ala-D/L-Glu racemase (EC 5.1.1.-)-Gt_Embl ## L-Ala-D/L-Glu racemase-Gt_GB	5.1.1.-	36.17	1.1	26.6
	E_coli							

The above procedure was applied to all remaining omitted genes and all of them were successfully found in our query strain of *G. thermoglucosidasius*.

3.2 Identifying misannotated enzymes

In contrast, the enzyme 5'-methylthioadenosine nucleosidase (EC 3.2.2.16) was found in the annotation of the query strain but not in the closely related reference organisms. There are two possible explanations for this: either *G. thermoglucosidasius* NCIMB 11955 has acquired an enzyme that its close relatives lack, or else this enzyme has been misannotated by one of the annotation servers used. Examining the PathwayBooster 'Annotations' and 'BLAST hits' sections, where no hits were found, we decided to look more closely at the gene encoding this enzyme. RTMO02286 has been assigned two potential functions: 5'-methylthioadenosine nucleosidase (EC 3.2.2.16) and S-adenosylhomocysteine nucleosidase (EC 3.2.2.9). Given that there were no hits found for EC 3.2.2.16 by PathwayBooster, our focus shifted to EC 3.2.2.9. This enzyme is assigned to all reference organisms and after examining the annotations and BLAST hits (Fig. 3.3), we concluded that EC 3.2.2.9 is the more probable annotation for RTMO02286.

(a)

EC Number	Species	genes	annotations
3.2.2.9	Gt_Ergo	RTMO02286	Gt_Embl
	G_thermoglucosidasius	Geoth_1095	Gt_KEGG
	G_kaustophilus	GK2542	Gk_KEGG
	G_thermodenitrificans	GTNG_2475	Gtn_KEGG
	G_WCH70	GWCH70_2476	Gw_KEGG
	G_Y412MC61	GYMC61_0977	Gy_KEGG
	B_subtilis	BSU27270	Bs_KEGG
	E_coli	b0159	Ec_KEGG

(b)

EC Number	Target Species	Target gene	Query gene	Query gene function	EC Number	Seq. similarity	e-value	blast score
3.2.2.9	B_subtilis	BSU27270	RTMO02286	## 5'-methylthioadenosine nucleosidase (EC 3.2.2.16) / S-adenosylhomocysteine nucleosidase (EC 3.2.2.9)-Gt_Embl ## 5'-methylthioadenosine nucleosidase-Gt_GB	3.2.2.16<br>3.2.2.9	66.23	8e-91	326
	E_coli	b0159	RTMO02286	## 5'-methylthioadenosine nucleosidase (EC 3.2.2.16) / S-adenosylhomocysteine nucleosidase (EC 3.2.2.9)-Gt_Embl ## 5'-methylthioadenosine nucleosidase-Gt_GB	3.2.2.16<br>3.2.2.9	53.91	3e-69	254

(c)

EC Number	Target Species	Target gene	Query gene	Query gene function	EC Number	Seq. similarity	e-value	blast score
3.2.2.9	B_subtilis	BSU27270	RTMO02286	## 5'-methylthioadenosine nucleosidase (EC 3.2.2.16) / S-adenosylhomocysteine nucleosidase (EC 3.2.2.9)-Gt_Embl ## 5'-methylthioadenosine nucleosidase-Gt_GB	3.2.2.16<br>3.2.2.9	66.23	8e-91	326
			RTMO00732	## Purine nucleoside phosphorylase (EC 2.4.2.1)-Gt_Embl ## Purine nucleoside phosphorylase-Gt_GB	2.4.2.1	26.56	9e-09	53.5
			RTMO02428	## DNA polymerase III alpha subunit (EC 2.7.7.7)-Gt_Embl ## DNA polymerase III alpha subunit-Gt_GB	2.7.7.7	37.50	0.053	31.2
	E_coli	b0159	RTMO02286	## 5'-methylthioadenosine nucleosidase (EC 3.2.2.16) / S-adenosylhomocysteine nucleosidase (EC 3.2.2.9)-Gt_Embl ## 5'-methylthioadenosine nucleosidase-Gt_GB	3.2.2.16<br>3.2.2.9	53.91	3e-69	254
			RTMO00732	## Purine nucleoside phosphorylase (EC 2.4.2.1)-Gt_Embl ## Purine nucleoside phosphorylase-Gt_GB	2.4.2.1	20.63	0.004	35.0
			RTMO03112	## NADH dehydrogenase family-Gt_Embl ## NADH dehydrogenase family-Gt_GB		25.93	0.39	28.1

Figure 3.3: PathwayBooster reports confirming the annotation of S-adenosylhomocysteine nucleosidase (EC 3.2.2.9) over 5'-methylthioadenosine nucleosidase (EC 3.2.2.16) for gene RTMO02286: (a) Annotations report, (b) Best bidirectional BLAST hit with *E. coli* and *B. subtilis*, (c) Top three BLAST hits.

Bibliography

Ashida H. *et al.* (2008) Enzymatic characterization of 5-methylthioribulose-1-phosphate dehydratase of the methionine salvage pathway in Bacillus subtilis. *Bioscience, biotechnology, and biochemistry*, **72.4**, 959-967.

Aziz R. K. *et al.* (2008) The RAST Server: Rapid Annotations using Subsystems Technology. *BMC Genomics*, **9**, 75.

Kanehisa M. *et al.* (2012). KEGG for integration and interpretation of large-scale molecular datasets. *Nucleic Acids Res.*, **40**, D109-D114.

Kanehisa M., Goto S. (2000) KEGG: Kyoto Encyclopedia of Genes and Genomes. *Nucleic Acids Res.*, **28**, 27-30.

Overbeek R. *et al.* (2003) The ERGO genome analysis and discovery system. *Nucleic Acids Res.*, **31**, 164-171.

Scheer M. *et al.* (2011) BRENDA, the enzyme information system in 2011. *Nucleic Acids Res.*, **39**, D670-D114.
